# JSim, an open-source modeling system for data analysis

**DOI:** 10.12688/f1000research.2-288.v1

**Published:** 2013-12-30

**Authors:** Erik Butterworth, Bartholomew E. Jardine, Gary M. Raymond, Maxwell L. Neal, James B. Bassingthwaighte

**Affiliations:** 1 Dept. of Bioengineering, University of Washington, Seattle, WA 98195, USA

## Abstract

JSim is a simulation system for developing models, designing experiments, and evaluating hypotheses on physiological and pharmacological systems through the testing of model solutions against data. It is designed for interactive, iterative manipulation of the model code, handling of multiple data sets and parameter sets, and for making comparisons among different models running simultaneously or separately. Interactive use is supported by a large collection of graphical user interfaces for model writing and compilation diagnostics, defining input functions, model runs, selection of algorithms solving ordinary and partial differential equations, run-time multidimensional graphics, parameter optimization (8 methods), sensitivity analysis, and Monte Carlo simulation for defining confidence ranges. JSim uses Mathematical Modeling Language (MML) a declarative syntax specifying algebraic and differential equations. Imperative constructs written in other languages (MATLAB, FORTRAN, C++, etc.) are accessed through procedure calls. MML syntax is simple, basically defining the parameters and variables, then writing the equations in a straightforward, easily read and understood mathematical form. This makes JSim good for teaching modeling as well as for model analysis for research.   For high throughput applications, JSim can be run as a batch job.  JSim can automatically translate models from the repositories for Systems Biology Markup Language (SBML) and CellML models. Stochastic modeling is supported. MML supports assigning physical units to constants and variables and automates checking dimensional balance as the first step in verification testing. Automatic unit scaling follows, e.g. seconds to minutes, if needed. The JSim Project File sets a standard for reproducible modeling analysis: it includes in one file everything for analyzing a set of experiments: the data, the models, the data fitting, and evaluation of parameter confidence ranges. JSim is open source; it and about 400 human readable open source physiological/biophysical models are available at http://www.physiome.org/jsim/.

## Introduction

The modeling of biological processes starts with defining the hypothesis to be tested in an experiment. To make scientific progress, Platt ( Platt, 1964) emphasized defining at least two distinct hypotheses and then designing an experiment with the power to clearly distinguish between these hypotheses. By so doing, at least one of the hypotheses must then be rejected: the rejection marks a stepping-stone in science. If a hypothesis is not rejected then it remains as a potential working hypothesis, the target of further experimentation that eventually will lead to its rejection or improvement.

The virtue of the *mathematically-defined* hypothesis is that it is clear and precise, and therefore susceptible to contradiction. Arguably, one should use mathematical “in numero experimentation” to define the critical laboratory experiment. Given that the experiment tests whether or not the working hypothesis is compatible with experimental data, then failure to fit leads to rejection. Revision of the conjecture follows: science is advanced.

The hypothesis testing cycle is an iterative procedure: design hypothesis (and alternative hypotheses) → execute experiment → evaluate goodness of fit of model to data → either reject the hypothesis and restart, or, alternatively, → accept the model as the current working hypothesis and assess the parameters for the specific situation. The working model serves as the current belief until deeper thinking leads to an alternative hypothesis and one restarts the cycle. This philosophical and procedural point of view, more or less guaranteed to make efficient progress in the field, creates definable results step by step, and gives investigators a sense of satisfactory success.

As in physics, models are posed in order to gain deeper understanding. Cause-and-effect models of biological systems are usually deterministic; they are fundamentally different from observationally-based probabilistic associations. The desire is to represent sequences of operations within a dynamic system leading to, and explaining, the observed data ( Coatrieux & Bassingthwaighte, 2006; Bassingthwaighte *et al.*, 2006a). Standard statistical methods are not central to deciding whether or not to reject the hypothesis, but are indeed very helpful in assessing goodness of fit, estimating confidence ranges and co-variances among parameters, and in guiding the investigator in identifying errors or in finding ways to simplify the model.

Over the years we have developed sets of tools to serve these processes. In this article we describe the features of a simulation analysis system, JSim; it is the product of evolutionary improvements in the hypothesis testing cycle. The central goals are to facilitate attempts to fit models to data, and to support the efficient development of computational models that describe and explain the behavior of biological systems ( Bassingthwaighte & Goresky, 1984; Bassingthwaighte *et al.*, 2005; Beard *et al.*, 2005).

Our perspective is embedded in JSim: it is an open-source simulation analysis platform, freely downloadable, running on Linux, Macintosh, and Windows, providing tools for the steps in the modeling analysis of data. There is a naturally occurring sequence of steps to take when one starts with an unanalyzed data set and has the goal of modeling the cause and effect relationships. We have found it useful to follow a simplified summary: The THIRTEEN STEPS:

## The THIRTEEN STEPS in the modeling process

These are proposed as a guide. The ordering is not rigid, but it is wise to cover all of the steps in one’s mind when starting and again when finishing up a study. Using the steps in the order listed here almost always works well.

(1) When starting with existing experimental data, plot and display the data so that one can rapidly review and compare multiple data sets. This also prepares for comparing with later model results.

(2) Develop the model, the mathematical formulation of the hypothesis. One may start with one or more existing models or modules of a similar nature (retrieved from a model repository or archival format) and modify it. Construct illustrations of model structure to aid the conceptual approach.

(3) Verify unitary balance in the model equations, an easy first check for model self-consistency.

(4) Select appropriate methods for solving model equations (e.g. differential equation solvers).

(5) Display model solutions graphically and in text listings. Inspect.

(6) Verify the mathematical accuracy of solutions. Check that results are not dependent on temporal or spatial step sizes, that mass or charge is appropriately conserved, and that limiting cases match analytical solutions.

(7) Explore model behavior over wide ranges of parameter values in state-space. (We think of “state space” as being the N-dimensional space enclosing the ranges of values of all of the parameters within which the model is correct numerically and sensible scientifically.)

(8) Perform sensitivity analyses, examining the fractional change in model solutions with fractional change in each parameter.

(9) Adjust parameters to fit model solution to data, manually or using an optimizer. Start from different places in parameter space and vary the optimization method to test solution uniqueness.

(10) Assess goodness of model fit to data. Plot residual differences to expose systematic biases.

(11) Examine parameter correlations to identify highly correlated parameters and reduce the number of free parameters in optimizations. Reoptimize.

(12) Evaluate parameter confidence ranges. The sensitivities at the “best fit”, expressed as the local curvature of the optimization cost function give a practical estimate. This can be refined using a Monte Carlo evaluation of parameter likelihoods as probability density functions.

(13) Preserve the source code, multiple data sets, multiple analyses and parameter sets, the settings (for initial and boundary conditions, parameter scans, displays, solver choices, optimizers, Monte Carlo, etc.), the graphs of results, the investigator’s notes and descriptions of procedures, plots, etc., all in a single, reproducible, exportable package. Share this package openly with collaborators, reviewers, and the public, a moral and perhaps ethical requirement when the support comes from public funds.

### Interpretation of analyses

What one wants primarily from modeling analysis is insight into mechanisms. JSim is efficient for model development and testing. The fitting of experimental data by model solutions does not provide proof that the model is correct. It says merely that the model can serve as a descriptor under limited range of circumstances, namely those examined in the experimentation. Validity is never provable. Likewise, causation may be identified, but deeper levels may exist to be revealed later.

What does the model predict? Every model, with a little ingenuity, can be queried. What would be the responses to different inputs? How would the system respond if a component were missing or damaged? Predictions then form the basis for the design of the next experimental test. Correct predictions, failing to invalidate the model, do strengthen the confidence in the model but only to the degree commensurate with the comprehensiveness of the particular prediction.

### Background

JSim is the latest in a series of modeling/data analysis programs dating back to SimCon ( Knopp *et al.*, 1970) (named for Simulation Control). SimCon provided a text and graphics interface to models written in Fortran. Between 1967 and 1993, the basic methods of data analysis (e.g. function generators, loops, sensitivity, optimization) were developed and refined within the SimCon framework. In 1993, SimCon was replaced by XSim ( King *et al.*, 1995), which implemented the same functionality under X-Windows on several Unix-like operating systems (SunOS, IRIX, Linux, AIX). XSim also added custom graphic model interfaces, on-demand expression graphing, worlds-within-worlds graphics ( Harris *et al.*, 1994), remote (client-server) computation and limited multi-processing. JSim development efforts began in 1999 and augmented the functionality developed in SimCon and XSim by adding simplified model specification (using the MML modeling language), facilities for data analysis and for distribution of results and of models (using project files), popular desktop and laptop support (Windows, Macintosh & Linux) and fully integrated multiprocessing for shared memory systems ( Raymond *et al.*, 2003).

### JSim overview

JSim is quite general, and while designed for evaluating models against experimental data, it also serves pure model development quite well. It is built around a “project file” (.proj), that may hold many data sets, several different models and the results of multiple types of analyses testing models against the data and against each other. JSim’s handling of ODEs (ordinary differential equations) suits it for traditional compartmental modeling and SBML ( Hucka *et al.*, 2003), CellML ( Cuellar *et al.*, 2003), and pharmacokinetic (PK) models in general. Solving PDEs (partial differential equations) hugely expands the range of processes that can be modeled in physiology and clinical medicine ( Goresky, 1963; Bassingthwaighte, 1974), biophysics, and PKPD modeling ( Roberts & Rowland, 1986). JSim handles spatial diffusion ( Barta *et al.*, 2000; Safford & Bassingthwaighte, 1977) and convection-diffusion problems. From soon after its release in 1999, JSim provided automated unit consistency checking in all equations and also automated unit conversion (such as minutes to seconds) in calculations ( Chizeck *et al.*, 2009). This pair of features automates the first stage of verification of the model’s mathematical implementation by making sure that every equation has unitary balance. Modeling taking account of the anatomical quantitative constraints is now recognized as critical and is facilitated by the automated unit checking ( Vinnakota & Bassingthwaighte, 2004). The second phase of compilation parses the details of the equations and sequences them for efficient computation. For an example, a cardiovascular-respiratory system model ( Neal & Bassingthwaighte, 2007), ran under JSim exactly 300 times faster than a Matlab-Simulink version of the identical model (Howard Chizeck/Stephen Hawley: personal communication).

MML (Mathematical Modeling Language) is the declarative modeling language developed for JSim and used for composing models. Its archival version is XMML, in the XML style of SBML and CellML. In MML, one writes mathematical equations directly into the code, and the MML compiler handles converting the set of equations into a sequence of computations. Since the equation representation is closely related to the conceptual formulation of the model, MML models are easily understood, and pieces of the model are readily interpretable as particular processes. The fact that one can write several models into a single MML program allows one to compare competing hypotheses (models). Having a standard layout for graphs and for ASCII text output of model solutions is convenient. For special purposes, as for a model to be used in clinical practice or teaching, an alternative graphical user interface specifically designed for the model can be readily substituted for the default layout. If a particular model absolutely requires procedural code, this can be developed in C, or Fortran or Java, and invoked as part of the model computation.

### JSim problem domain

JSim is a general purpose simulation and data analysis software system. It handles a wide range of mathematical problems including algebraic equations, ordinary differential equations, and parabolic, hyperbolic and elliptic partial differential equations. It contains 8 ODE and 3 PDE solvers implementing a variety of algorithms which allow the flexibility to strike a balance between accuracy and computational speed. It performs time series analyses including forward and backwards Fourier transforms. MML can handle multi-dimensional PDEs but the solvers currently implemented support only two dimensions (typically time and one spatial dimension). For two spatial dimensions the problem needs to be formulated into either ODE nodes or PDEs in one spatial dimension linked by ODEs in the other spatial dimension. JSim does not support complex numbers or matrix notation and associated matrix operators; in JSim all matrices must be written explicitly as a set of equations.

JSim can be used in any discipline where mathematical equations are used for modeling and analyzing data. JSim was originally developed to model and analyze physiological phenomena and many of the built-in tools were developed to handle physiological problems. But all of the JSim tools can be applied to any other scientific discipline. JSim excels at analyzing time course and spatial domain data in complex systems ( Beard & Bassingthwaighte, 2000; Beard *et al.*, 2005; Bassingthwaighte *et al.*, 2006b; Suenson *et al.*, 1974; Safford & Bassingthwaighte, 1977). Examples include modeling pharmacokinetic/dynamic (PK/PD), radiological (CT, PET, MRI) and multiple indicator dilution (MID) data.

### JSim’s Mathematical Modeling Language, MML

JSim uses the Mathematical Modeling Language (MML) to describe models. When JSim imports other model formats (e.g. SBML, CellML, Antimony), it translates them to MML. MML is a concise, ASCII text language for defining parameters and variables and for writing the equations describing a model. MML is a declarative language (as opposed to procedural or imperative languages such as MATLAB, Java, Python, and FORTRAN), meaning that, in MML, equations represent mathematical equality, rather than providing a directive to calculate the left-hand side variable from the expression on the right. In MML, it makes no difference if terms in an equation appear on the left or right hand side. Such equations are a direct representation of the mathematical ideas in a model rather than a procedural formulation. This improves readability and allows for more extensive consistency checks than procedural formulations. The MML compiler checks to ensure that all variables are completely, but not overly, specified – a check unavailable in procedural languages. The compiler sequences the calculations based on the dependencies of the variables to be computed, thus eliminating order-of-operations errors that are possible in procedural languages. MML variables are (optionally) labeled with physical units, enabling the compiler to reject equations with unitary imbalances; this also allows the automated insertion of appropriate unit conversion factors when needed ( Chizeck *et al.*, 2009) (e.g. mmHg to kPa). This relieves the modeler of the burden of adding unit conversion factors (another potential source of error) and aids readability, since equations need not be cluttered with conversion factors. MML’s design supports the model development and unit balance aspects of modeling steps 2 and 3 above. An example of MML code is shown below as Box 1, which codes a “progress curve”, the concentration-time curves for hypoxanthine to xanthine to uric acid catalyzed by the enzyme xanthine oxidase through the two oxidation steps. MML code for partial differential equations is given in Box 2.

Box 1. **Model code for a reaction sequence (Model #320 at www.physiome.org).**

// Model Name: MM2irrev (From reference JBBass13, data of Escribano ( Escribano *et al.*, 1988))

/* Brief Description: The “MM2irrev” program codes a sequential pair of irreversible Michaelis-Menten enzymatic reactions, Hx → Xa → Ua, wherein the one enzyme, xanthine oxidase, serves both steps. Hx and Xa compete for its active site. */

import nsrunit; unit conversion on;

math MM2irrev {

realDomain t sec; t.min=0; t.max=5000.0; t.delta=1.00; // t is independent variable

// PARAMETERS: (denoted param(t) if time-variable) (all changeable at run-time)

     real Vhmax = 1.84 uM/s;       // Vmax for enzymatic conversion of Hx -> Xa

     real Kmh = 3.67 uM;            // Km for assumed instant binding of Hx to enzyme

     real Vxmax = 1.96 uM/s;      // Vmax for Xa -> Ua

     real Kmx = 5.94 uM;          // Km for assumed instant binding of Xa to enzyme

     real Hzero = 46.3 uM, Xzero = 0 uM, Uzero = 0 uM; // initial conditions

// VARIABLES (specified as functions of time by (t) appended in defining the name)

     real H(t) uM;          // concentration of Hx (HypoXanthine)

     real X(t) uM;          // concentration of Xa (Xanthine)

     real U(t) uM;          // concentration of Ua (Uric acid)

// INITIAL CONDITIONS (t.min can differ from t = 0 sec.)

     when (t=t.min){ H= Hzero; X = Xzero; U = Uzero;}

// SYSTEM OF EQUATIONS (3 ODEs) (Derivative dH/dt written as H:t)

    H:t = - (Vhmax*H/Kmh) / (1 + H/Kmh + X/Kmx);                                      // Hx→Xa

    X:t = ((Vhmax*H/Kmh) - (Vxmax*X/Kmx)) / (1 + H/Kmh + X/Kmx);    // Xa→

    U:t = (Vxmax*X/Kmx) / (1 + H/Kmh + X/Kmx);                                      // →Ua

} // PROGRAM END

### Numeric solvers

MML is designed without reference to the numerical algorithms that will be used for simulation. Rather, the user selects the numerical methods in the JSim run time user interface. At present JSim provides 8 algorithms for solving ODEs ( Table 1) and 3 for PDEs ( Table 2). Numerical methods for stochastic simulation are variants on the Gillespie algorithm ( Gillespie, 1977). JSim’s solvers support modeling steps 4 to 6 above.

**Table 1.** JSim ODE solvers.

Auto	Starts with Dopri5, if Dopri5 fails, switches to Radau
Dopri5	Dormand-Prince explicit Runge-Kutta method of order 5(4) for non-stiff equations ( Hairer *et al.*, 1993)
Radau	Implicit Runge-Kutta method (Radau IIA) of variable order (switches automatically between orders 5, 9, and 13) ( Hairer & Wanner, 1996)
KM	Five stage, 4th order accurate Merson-modified Runge-Kutta method with adaptive steps ( Merson, 1957)
Fehlberg	Fifth order accurate Runge-Kutta-Fehlberg Method with adaptive stepsize, also known as RK45 ( Fehlberg, 1969)
Euler	Explicit forward Euler Method, first order accurate ( Euler, 1768–1770; LeVeque, 2007)
RK2	Two-stage explicit Runge-Kutta method, 2nd order accurate ( LeVeque, 2007)
RK4	Classical Runge-Kutta explicit 4th order four-stage method ( LeVeque, 2007)
CVode	CVODE, a publicly available stiff ODE solver ( Cohen & Hindmarsh, 1995)

**Table 2.** JSim PDE solvers.

LSFEA	Lagrangian Sliding Fluid Element Algorithm ( Bassingthwaighte, 1974; Bassingthwaighte *et al.*, 1992; Poulain *et al.*, 1997). The convecting step is solved separately from the other processes
MacCormack	2nd order accurate finite difference method for solving hyperbolic differential equations ( MacCormack, 1969)
TOMS731	Finite element discretization akin to a nonlinear Galerkin method 2nd order accurate in space ( Blom & Zegeling, 1994)

To solve differential equations one needs initial conditions, and JSim’s parser (precompiler) demands these, as in Box 1. Partial differential equations require also boundary conditions, as seen in the code for a two-region convection-diffusion-permeation-reaction model ( Box 2).

### Function generators

Many physiological systems or components (e.g. one for the uptake of a metabolite) can be considered as operators. The operator takes an input function (e.g. inflowing solute concentration) and produces an output function (e.g. outflowing solute and metabolite concentrations). Model behavior can be tested by using various input waveforms (e.g. as in Box 2 “extern real Cin(t)”) described by JSim “function generators”. These might be time series signals of diverse form (pulses, pulse combinations, sines, shaped sawtooth), probability density functions (Gaussian, exponential, Poisson, lognormal, gamma variate, random walk, etc.), or come directly from experimental data. When the system is linear (output area equals input) and stationary (response same at another time), then the output is the convolution of the operator’s transfer function (the response to an infinitely short pulse input) with the input function. Users select input functions at run time for testing numerical algorithms for correctness (verification testing), for model exploration (behavioral analysis) or for analyzing data as for steps 6 and 7 in our “13-Step” process.

### Model behavioral analysis and visualization

**Figure 1.**
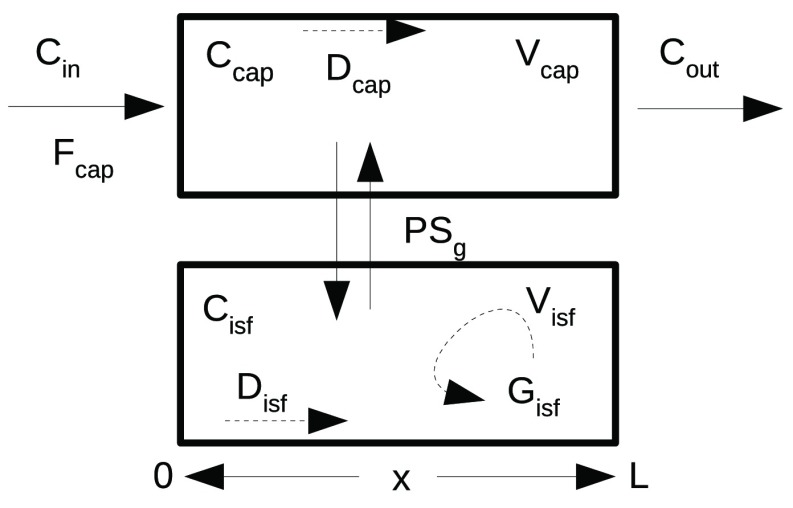
Capillary-tissue exchange unit. Fluid flows with velocity F _cap_*L/V _cap_ along the capillary from the entrance at x = 0 to the exit at x = L, and exchanges across the capillary wall into a stagnant extravascular region with conductance PS, the permeability-surface area product. The input is a bolus of solute, C _in_(t), entering the capillary with the flow, F _cap_. Axial gradients along the capillary are diminished by diffusion, D _p_ and D _isf_. Tissue consumption occurs at rate G _isf_*C _isf_. This is a simplified version of models used for indicator dilution studies and PET clinical studies ( Beard & Bassingthwaighte, 2000; Bassingthwaighte *et al.*, 1989; Bassingthwaighte *et al.*, 1992; Bassingthwaighte *et al.*, 2006b).

We will use a simple convection-diffusion reaction model ( Bassingthwaighte, 1974; Bassingthwaighte & Goresky, 1984) to illustrate some facilities for visualizing model solutions and the effect of varying parameter values on them. The system is diagrammed in Figure 1 and the code is provided in Box 2.

Box 2. **Code for a 2-region Blood-Tissue Exchange Model.**

// MODEL NUMBER: 0190 at www.physiome.org ( Bassingthwaighte, 1974)

// MODEL NAME: BTEX20simple

// SHORT DESCRIPTION: Simple Model of an axially distributed two-region

//  capillary Blood-Tissue EXchange unit with consumption in interstitium

import nsrunit; unit conversion on;

math btex20simple {

//  INDEPENDENT VARIABLES:

realDomain t sec ; t.min = 0; t.max = 30; t.delta = 0.1;

realDomain x cm; real L= 0.1 cm, Ngrid = 31; x.min = 0; x.max = L; x.ct = Ngrid;

// **Parameters and Keys to Names:**

real Fcap   = 1 ml/(g*min),   // Capillary (cap) plasma flow

   Vcap   = 0.05 ml/g,           // Capillary Volume

   Visf    = 0.15 ml/g,              // Interstitial Fluid (isf) Volume

   PS   = 1 ml/(g*min),           // Permeability-surface area product: cap <--> isf

   Gisf    = 0 ml/(g*min),          // consumption rate in isf region (Gulosity)

   Dcap   = 1.0e-5 cm^2/sec, // cap axial diffusion coefficient

   Disf   = 1.0e-6 cm^2/sec; // isf axial diffusion coefficient

// **Inflow Concentration, Input Function:**

extern real Cin(t) mM;

// **Concentration Variables:**

real Ccap(t,x) mM,       // capillary concentration at position x

     Cisf(t,x) mM,      // isf     concentration at position x

     Cout(t) mM;      // Outflow Concentration from capillary at x=L

// **Boundary Conditions:** (Note total flux BC for inflowing region.)

when (x=x.min) { (-Fcap*L/Vcap)*(Ccap-Cin)+Dcap*Ccap:x = 0; Cisf:x = 0; }

when (x=x.max) { Ccap:x = 0; Cisf:x = 0; Cout = Ccap; } // reflecting boundary

// **Initial Conditions:**

when (t=t.min) { Ccap = 0; Cisf = 0; } // sets initial concentrations to zero

// **Partial Differential Equations:** Ccap:t is dCcap/dt in JSim’s MML (ODE or PDE)

   Ccap:t = -Fcap*L*Ccap:x/Vcap + Dcap*Ccap:x:x + PS*(Cisf-Ccap)/Vcap; // dCcap/dt

   Cisf:t = -Gisf*Cisf/Visf              + Disf*Cisf:x:x     + PS*(Ccap-Cisf)/Visf;  // dCisf/dt

}    //program end

### Plot pages

JSim provides several mechanisms for visualization, providing insight about model dynamics. The most basic are plot pages, each of which may contain line, scatter, contour and colormap plots. One may plot experimental data and model solutions (from one or more models), scaled automatically or manually, linear or logarithmic, plotted as they are being computed or displayed or edited later. Multiple plot page configurations are stored in each project, enabling reproducible analysis (e.g. all the data and graphs for a particular journal article). JSim plot pages support modeling steps 1, 5, 6, 7, 8 and 10 above (display of experimental data and model solutions, verify solution accuracy, explore model behavior, display of sensitivity curves and assessments of goodness of fit).

### LOOPS: Iterating solutions to exhibit behavior

**Figure 2.**
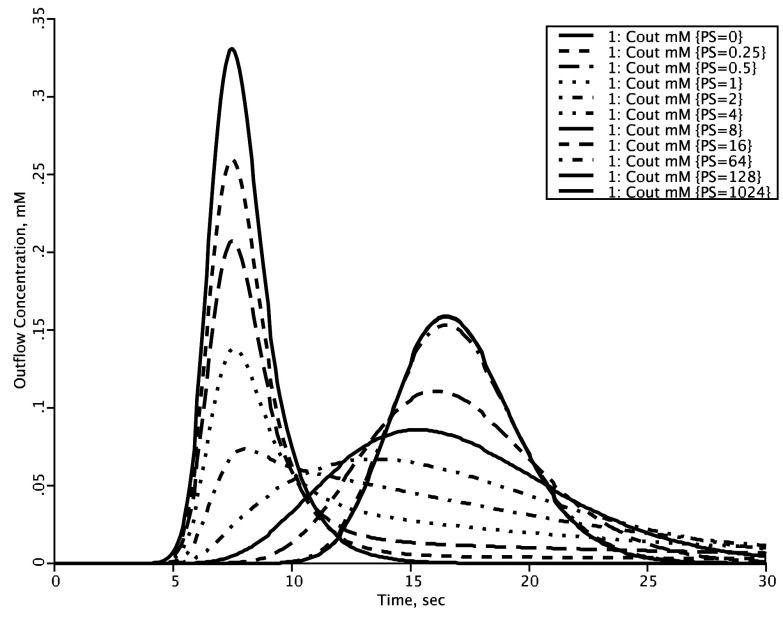
Using LOOPS to explore parameter influences. With the permeability surface area product (PS) = 0 (taller solid curve) the outflow concentration-time curve, C _out_, represents the response function via the vascular space alone. The mean transit time for this is V _cap_/F _cap_. With finite PS there is extraction of solute during transcapillary passage, shown by the successive diminutions of the heights of the initial peaks as PS increases. At low PSs the form of the outflow starts as a reduced version of the curve with PS = 0; in this state the flux into the tissue is purely "barrier limited". When PS is 4 or greater ml/(g*min), the sixth curve, the initial peak is no longer discernable; at yet higher PSs a second peak arises, and at PSs above 128 ml/(g*min) increasing the PS further has no effect on the shape of the outflow curve; in this state the exchange flux is purely "flow-limited", where changing the flow shifts C _out_, but changing PS does not.

Model loops are a feature for behavioral analysis that plot data from a family of model runs using a user-chosen sequence of parameter values. For example, Figure 2, “looping over”, i.e. making a sequence of changes in a parameter value for the membrane permeability in a tracer uptake model yields a family of plots showing how outflow tracer concentration curves would vary with varying permeability. The curves, of course, depend upon the settings for the other parameters of the model, so the looping sequence should be initiated under widely divergent conditions in order to understand the “conditions” (the regions of state space) where the chosen parameter may have little influence or maximum influence. JSim’s loops facility support modeling steps 6 and 7 above (verify solution accuracy, explore model state space). A convenient feature of the LOOPS function is that the user can stop the solution, automatically starting the next one, whenever desired, speeding up the review of solutions. This is especially important in large models with long computation times.

### Nested plots

**Figure 3.**
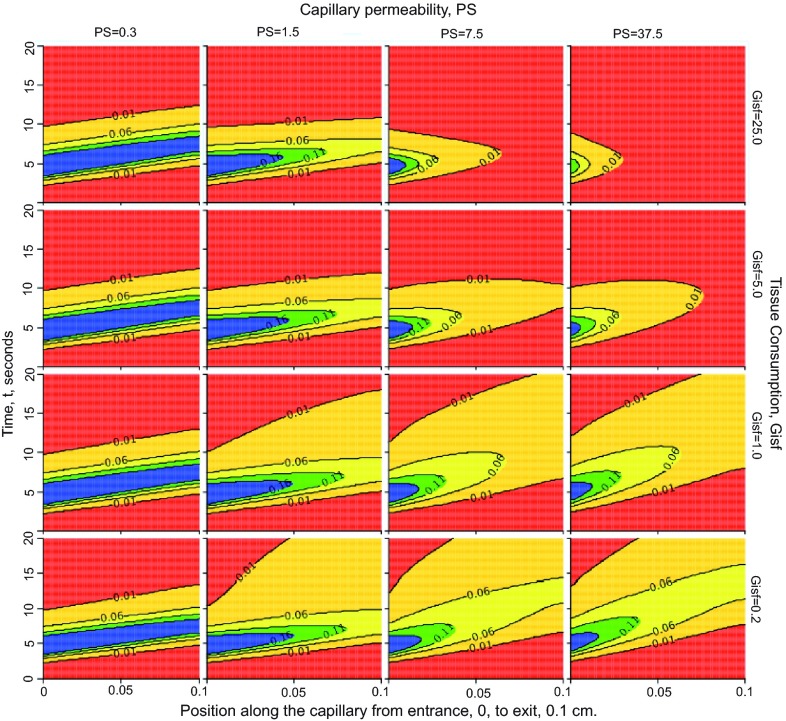
Nested plots. Behavior of the two-region model when varying capillary permeability, *PS,* and tissue consumption, *G*_isf_. Each panel is a contour plot with the position between the capillary entrance at *x*=0 to the exit at *x*=0.1 cm on the abscissa, and time, *t*, on the ordinate. At each time step (ordinate) the horizontal line from 0 to 20 is colored (using color profile “rainbow” in this case) in accord with the concentration at each point in *x*. Convection moves the entering solute along the tube from left to right to larger *x* on this graph. With successive times the colored horizontal lines construct a shaped profile above the *x-t* plane; contour lines with units in mM are superimposed. The columns from left to right show contours with *PS* increasing by factors of 5 (see labels at top of column) from *PS* = 0.3 to 37.5 ml/(g*min). The consumption *G*_isf_ increases from 0.2 in the bottom row by factors of 5 to the top row with *G*_isf_ = 25 ml/(g*min); see labels on right ordinate. With low *PS*, leftmost column, very little of the solute escapes into the tissue, so the injected bolus remains relatively compact even while undergoing some diffusional spread ( *D*_cap_ = *D*_isf_ = 10 ^-4^ cm ^2^/sec), and the influence of the consumption is negligible since so little enters the ISF. With increasing *PS* more solute enters the ISF where it is consumed. With high *PS* and high *G*_isf_, the right uppermost panel, the solute is all consumed before it can reach the capillary exit at the right edge of the panel. [This plot is set up under “Project”, “Add”, “New Nested Plot” using LOOPS, inner and outer, to set the values for the parameters, and on the NestedPlot, then clicking on “XY plot” to choose “contour”. Instructions are under Running JSim – Data Analysis – Nested plots: www/physiome.org/jsim/docs/User.html].

Nested plots ( Figure 3) are JSim’s version of worlds-within-worlds graphics ( Harris *et al.*, 1994). Each nested plot is a 2-dimensional array of plots, each of which represents the form of a set of model solutions with a pair of distinct parameter value. Nested plots enable simultaneous visualization of the effect of up to six independently varying parameters. JSim nested plots support modeling steps 6–8 above (verify accuracy, explore model state space, sensitivity analysis).

### Sensitivity analysis

**Figure 4.**
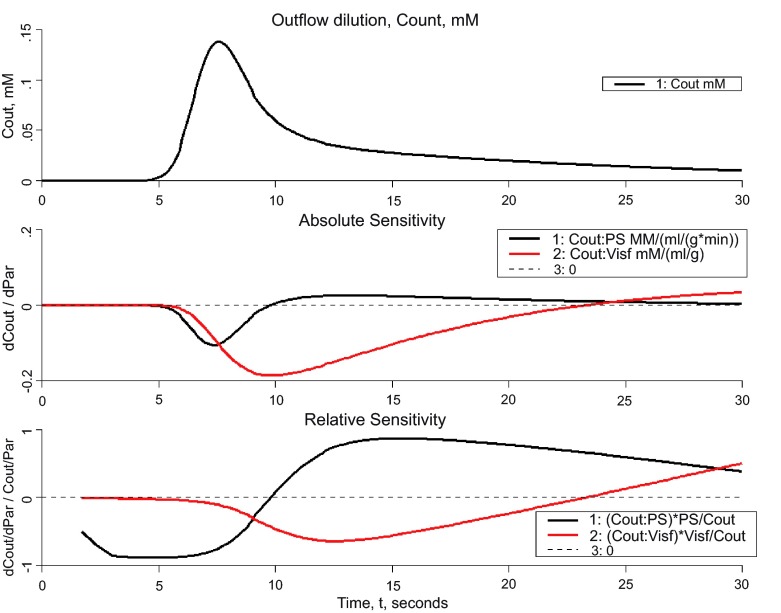
Sensitivity analysis using the same model as in Box 2 and Figure 2 and Figure 3. *Upper panel*: Model solution for outflow from capillary. Parameters were as in Box 2, the default parameters. *Middle panel*: Sensitivity function, *df/dp*, the change in C _out_ with a 1% increase in the capillary wall conductance (PS), black curve or the interstitial volume (V _isf_). *Lower panel:* Normalized sensitivity function, ( *df/f)/(dp/p)*, the fractional change in C _out_ divided by the fractional change in each parameter, again for a 1% change in the parameter value.

By “sensitivity analysis” we mean the examination of the influences of individual parameters on the model responses under a wide variety of conditions. The sensitivity function, S(t) is the change in magnitude, dQ, of variable Q, to a small change in a parameter value, dP. It may be expressed in a normalized form, (dQ/Q)/(dP/P), or unnormalized form, dQ/dP. As an example consider the same model as was explored in Figure 3. Figure 4 shows the sensitivities of the outflow concentration of a solute to a change in interstitial fluid volume (V _isf_) or capillary wall conductance (PS) following an injection of that solute at the capillary entrance. The upper panel shows the outflow concentration without parameter perturbation. The middle panel plots the unnormalized sensitivity functions, and the bottom plot shows the normalized sensitivity functions (with the early part of the curves removed when C _out_ is negligible). Increasing PS will lower the height of C _out_ for the first 10 seconds with the greatest reduction at the peak of C _out_ at ~8 seconds (due to greater flux of metabolite into the ISF); after 10 seconds, the height of C _out_ will be increased (back flux of metabolite from ISF). Increasing V _isf_ has the effect of lowering C _out_ for the first 24 seconds, then raising it after 24 seconds. JSim’s sensitivity analysis supports modeling step 7 above.

### Optimization

Manual parameter adjustment to fit the model to experimental data is encouraged as a means of gaining insight into model behavior. Automated parameter optimization is usually much faster; eight methods are provided (See Table 3); we recommend testing several in order to test speed and reliability with respect to the particular types of data and model. Given that some parameters are known or highly constrained, one may obtain the best model fit to the data for a particular subset of model parameters, and one may also, for some but not all of the optimizers, constrain the range for each parameter value, applying scientific judgment. Optimization helps in finding systematic misfits to the data (and the possible rejection of the hypothesis), and in estimating parameter values.

**Table 3.** JSim’s optimizers.

Simplex	A bounded, non-linear steepest-descent algorithm ( Dantzig *et al.*, 1995)
GGopt	Derivative-free non-linear optimizer. Uses adjustable mesh and linear least squares to find smoothed values of function, gradient and Hessian at center of mesh. Values drive a descent method that estimates optimal parameters, but is unbounded ( Bassingthwaighte *et al.*, 1988)
GridSearch	A bounded, parallel algorithm. Operates via progressively restricted search of parameter space on a regularly spaced grid of N points per dimension ( Kolda *et al.*, 2003)
NelderMead	Unbounded, steepest descent similar to Simplex ( Nelder & Mead, 1965)
NL2SOL	An adaptive nonlinear least-squares algorithm ( Dennis *et al.*, 1981; Dennis & Schnabel, 1983). Unbounded
SENSOP	A weighted nonlinear least squares optimizer using a variant of the Levenberg-Marquardt method to calculate the direction and the length of the step change in the parameter vector ( Chan *et al.*, 1993). Bounded
SimAnneal	Simulated annealing for finding the global optimum of a function in a large multi-dimensional parameter search space which is first randomly sampled with step-size decreasing with time ( Kirkpatrick *et al.*, 1983)
Genetic	Genetic algorithms are a family of algorithms that generate a population of candidate solutions selecting the best solutions in each iteration to “mutate” and “cross over”, creating a new generation of solutions in an iterative process. ( Holland, 1992)

The optimizer works to minimize an objective function, usually a weighted sum of squares of the differences between the model solution and the experimental data at each observation time or spatial position. This may require freeing up most parameters for optimization to make sure that an assumed constraint isn’t creating a biased solution. JSim provides a graph of residuals (the differences between model and data); sign tests and other statistical appraisals of the residuals as a function of time help to distinguish systematic from random deviations. JSim’s optimization facilities support modeling steps 9–12 above (fitting solutions, assessing goodness of fit, examining parameter correlations, evaluating confidence limits).

### Parameter confidence ranges

Model fitting to the data is never unique but is guided by the weighting of the observed data points and the noise in the data. Parameter estimates are not exact, but merely estimated, and even possibly biased by the user’s choice of the weights on individual data points. How to obtain a “best fit” of model function to data is always, in a sense, a personal choice. Guidelines include weighting inversely to the likely standard deviation of each data point, or unweighting outliers. Viewing the graph of residuals (the differences between data and model) is most helpful in identifying systematic misfits.

Ignoring how one got to the point of “best fit”, one desires an evaluation of the parameter values. If the optimized parameters do generate outputs that closely match the experimental data, the question becomes what confidence can be placed on these estimates. One simple method is to optimize using several different numerical method, i.e. different optimization algorithms and different weighting schemes, to see how much the “best fit” parameter estimates change. Other methods of estimating parameter confidence limits include using the Jacobian and using Monte Carlo methods.

**Using the Jacobian**: The Jacobian matrix is the matrix of the sensitivity functions for all the parameters open to optimization, as calculated at the location of the minimized objective function, the “best fit”. This matrix, which JSim calculates after each optimization provides the basis for determining correlations among parameters, and the confidence limits (standard deviations and expected ranges based on Gaussian assumptions). The calculation assumes symmetry and linearity, and so makes only local calculations, and gives no guarantee that the “best fit” is a global best fit. While getting to the “best fit” point in parameter space is data-dependent, this confidence range estimation procedure is not at all, for it is estimated solely from the shapes of the local sensitivity functions. Thus it behooves one to get the differing estimates obtained from different optimizers, different numbers of parameters searched, and even to move the parameter “best fit” values a little away from the optimizer’s choice and recalculate the confidence ranges.

**Using a Monte Carlo method**: A more robust, but more demanding, confidence limit calculation uses Monte Carlo methods. The procedure is to 1) Select a noise profile for each experimental data point, ideally based on what you believe the real noise is, e.g. 5% proportional Gaussian random noise. 2) Generate a perturbation for each experimental data point by drawing randomly from the selected noise profile. 3) Re-optimize the model against the new set of perturbed data points to obtain another estimate for each parameter. 4) Repeat steps 2 and 3 many times (e.g. 1000). From these results, one obtains a histogram of estimates for each optimized parameter, and robust confidence limits can be drawn directly from these histograms without assuming symmetry and linearity as in the Jacobian method. JSim displays these histograms to show the distributions of parameter estimates in full detail, and 2-parameter scatter plots to show covariance. (JSim’s confidence limit calculations support modeling step 12 above.)

### Network graphs

**Figure 5.**
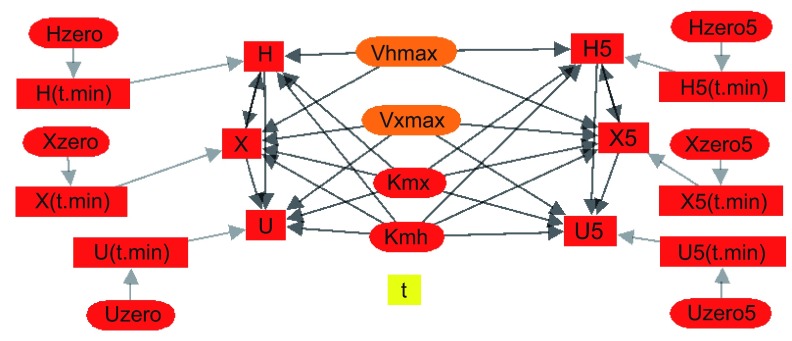
Connectivity graph for a modified version of the model program in Box 1. For Hx→Xa→Ua, the oxidation of hypoxanthine to xanthine to uric acid, catalyzed by xanthine oxidase. The connectivity is shown for a dual solution version of the code for fitting two different sets of experimental data simultaneously with a common group of parameters so as to obtain a minimally biased set of parameter confidence ranges.

JSim’s model “browser” provides a visual representation of model variables as “nodes” and their dependencies or connectivity with each other as connecting lines or “edges”. See Figure 5. The graphs can be selected to include model parameters, or selected classes of variables, e.g. pressures, strains, concentrations. This capability is based on work by Yngve ( Yngve *et al.*, 2007). JSim’s model browser supports modeling step 2 above (development of the model).

## Implementation

JSim is implemented in the Java computer language ( Gosling & McGilton, 2003). The major factors affecting this choice are Java's platform independent GUI (allowing Windows, Macintosh and Linux versions to be developed in a single code base), object-oriented features and garbage collection (simplifying complex coding), advanced utilities (associative arrays, dynamic linking, remote procedure calls), strong type checking (allowing many common coding errors to be caught at compile time) and robust exception mechanism (simplifying coding and enabling a virtually crash-proof GUI). Native code (C and Fortran) is used in certain restricted circumstances using the Java Native Interface (JNI) ( Liang, 1999) to reduce computational overhead (transcendental functions, 2D array access) and the availability of legacy code libraries (ODE, PDE and optimization numerical methods).

The MML language is parsed using JLex scanner generator and the CUP parser generator ( Appel, 1998). These tools, similar to the classic Unix lex and yacc ( Aho *et al.*, 1988), were among the few parser generation tools available for Java when JSim was first developed. Using a formal parser generator allows MML to be concise, intuitive, consistent and extensible. MML's declarative structure is an intuitive expression of a model's underlying mathematics (simplifying the modeler’s learning) and allows the overall structure of the model to be examined for mathematical correctness (detecting overspecification or underspecification) in a way that is not possible with a procedural specification. Units and unit checking ( Chizeck *et al.*, 2009) were added to MML soon after its initial design to further improve model conciseness and assure unit balance in the equations as a first step in verifying that the mathematics is rendered correctly by the numerics.

MML is compiled into Java model computational code for run-time execution. This results in faster model execution (in comparison to table-driven computations) and allows more flexible model computational structure (multiple time sweeps, indexed loops). JSim models run asynchronously to the GUI in contrast to most simulators which alternate computational and graphical update steps. This approach dramatically improves performance and user response, especially when remote computation is used. JSim's remote computation is implemented using Java Remote Method Invocation (RMI) ( Harold, 1997), providing reliable access to networked computational servers. This approach also isolates the JNI methods (above) in the computational engine, allowing the JSim GUI to run as a pure Java browser applet. JSim multiprocessing is implemented using Java threads ( Oaks & Wong, 2004) providing excellent performance and seamless integration with the Java memory management and exception mechanisms (providing application stability). MML code is stored as XMML for distribution, and has automated translators into XMML, SBML, CellML, and with limitations into Matlab ( Smith *et al.*, 2013).

## Reproducibility

The issue of reproducibility, or should we say the all-too-frequent failures of attempts to reproduce published results, are beginning to be recognized as a critical problem in advancing the biological sciences. It is easy to understand biological studies, with inherently great variability in materials and analysis procedures, should be less exact than those in the physical sciences, but it is not so forgivable that reproducing mathematical models of biological systems is a *major* problem. The two major repositories of published biological models, Biomodels ( http://www.ebi.ac.uk/biomodels-main/) using SBML ( www.sbml.org) and CellML (models.cellml.org), together have about 1000 curated models: there were errors in the publications requiring corrections in all but 5 of these, before the models could be demonstrated to run appropriately. These models all used algebraic, ODEs, or differential-algebraic equations and so must be regarded as relatively simple computationally compared to finite-element models or spatially dependent models. That only 0.5% of the not very complex models were reproducible is truly alarming, and demonstrates the lack of dedication to making scientific advances useful to others. Some open access journals, such as F1000Research, are aiming to improve this sad state, by requiring open source code to be deposited, hopefully along with the data that provide tests of the model hypotheses. A Special Section in *Science* ( Stone & Jasny, 2013) is devoted to the issues of open access, addressing open access, peer review, the changing publishing scenario, and encouraging broader methods of communication. F1000s founder, Victor Tracz, is featured as the “Seer of Science Publishing”, prodding us to do better.

### Project files

JSim project files store a set of codes for models, illustrative figures or diagrams, parameter sets, multiple data sets, the settings for looping, sensitivities, behavioral analysis, and optimizations, plot page configurations, and for project notes. Many models in the Physiome Repository (most of which are JSim-based) have experimental data in the project files for validation testing. Project files support the reproduction of a set of simulations and analyses for their sharing across JSim’s supported platforms (Windows, Mac OS, Linux). Project files support the modeling steps 1 and 13 above (from importation of data, to preservation and distribution of analyses). The MML, XMML and all the data and analyses are preserved in an ASCII format; thus the files tend to be small. The models described above take < 100 kB; large models with several hundred ODEs take up < 500 kB even with large time series of physiological data. These files are all human readable, and ready to run when opened in JSim. They contain everything used by the program: the notes, the source code, and the control parameters for all the steps in the analysis. They are editable in any word processor, but one avoids doing that since it is easier to enter code and notes under JSim and not risk disturbing the format in the XMML file that JSim reads.

 There are many models on the Physiome Repository ( www.physiome.org) with multiple data sets, model fits to data, and optimization results. Examples are that of Kuikka *et al.* on glucose uptake by myocardium ( Kuikka *et al.*, 1986), [models 163 and 173], xanthine oxidase reactions ( Bassingthwaighte & Chinn, 2013), [model 324], and lung endothelial serotonin uptake ( Jardine & Bassingthwaighte, 2013), [model 198]. All the JSim project files are stored in a Concurrent Versions System (CVS) archive so that the latest versions, as well as older versions, are always available. The models themselves are copyrighted but researchers are given the freedom to download, modify, and to construct new models from them so long as original authorship is acknowledged.

### Modeling over the web

The archived JSim models at www.physiome.org can be run over the web, with complete freedom to vary the parameters, modify the code, compile and run, import one’s own data for analysis, and save a modified and augmented file to one’s own computer for further use. (Models based on MATLAB or FORTRAN, a small fraction of the repository, cannot be run over the web but can be downloaded).

## Summary

JSim is a tool for hypothesis exploration and for analyzing data. Many of the steps in data analysis are built into JSim. It’s declarative modeling language, automatic unit balance checking, and built-in tools for solving ODEs, PDEs, and implicit equations greatly facilitate generating mathematically and physiologically consistent models. The built-in optimizers and associated statistical data reporting, along with behavior tools, such as parameter looping and sensitivity analysis, allow one to verify and explore model behavior in the context of experimental data and simulated data from previous models. With the ability to save these model ‘explorations’ as parameter sets within the JSim project file anyone can easily create a modeling and data analysis package that is easy to reproduce and distribute to others.

As a research tool, JSim has been developed and refined to accelerate the processes of modeling and data analysis. Adherence to quality standards augments efficiency ( Smith *et al.*, 2007). The time savings don’t simply reduce the time necessary to get to a result, they also end up improving the quality of the science in two ways. First, when it only takes a few seconds to tweak a model, re-run it, and view the results, researchers are more likely to explore many “what if” scenarios and develop a deeper understanding of model behavior, and in turn, a deeper understanding of the system being modeled. Second, researchers are more likely to do better verification checks and higher-level analyses if they are easy to do. When a few mouse clicks are all it takes to change solvers, time step sizes, optimization parameters, or even perform a complex Monte Carlo analysis to assess parameter correlations and confidence intervals, researchers are more likely to actually do those critical numerical checks and to take the model analysis beyond simply reporting a single parameter value.

In addition to its use as a research tool, JSim is also very useful as a teaching tool. JSim has been used in classes for high school, undergraduate, and graduate students, as well as many workshops for faculty members. The fact that JSim is open source, quick to download and install, as well as executable over the web, means that it is easily available to students. The simplicity of JSim’s model specification language, where users can focus on writing and working with the mathematical equations themselves rather than controlling program flow, means that students with no programming experience can rapidly begin to understand, create, and modify JSim models. Furthermore, JSim’s interactive plotting interface and the easy access it provides to sophisticated analysis tools such as sensitivity and Monte Carlo analysis allow students to perform analyses which would ordinarily be too difficult and time consuming for them to do on their own.

## Future developments

### Modular modeling

JSim has provided support for modular modeling from its inception ( Bassingthwaighte, 2000) using both mathematical and biological approaches, but now, with the developing recognition that models are more consistently understandable and more amenable to modular construction when they are annotated using identified ontology systems, libraries of modules present great opportunity for efficient construction of complex model systems. A module can be thought of as a self-contained model of an element of the larger system model and represents a specific physical, chemical or phenomenological process. A model might use multiple instances of the same module, for example, differently parameterized Michaelis-Menten type enzymatic reactions used for different reactants. One can build large models from a variety of modules representing physical or chemical processes such as the flux via a cell membrane transporter or ion channel or an enzymatic reaction, or a transcription regulatory pathway ( Beard *et al.*, 2005) incorporating knowledge of their connectivities. Allowing the modeler to draw pre-existing modules from a repository or extract them from previously developed models and enables the modeler to create new models quickly for hypothesis testing, a key to Physiome development ( Bassingthwaighte *et al.*, 2009). Below are two approaches to implementing modular modeling within JSim.

**Modular Program Constructor (MPC):** MPC focuses on using easily understood directives to extract generically coded JSim MML equations from files, changing the names of the generic variables to ontologically informative names and assembling the resulting code into new equations ( Raymond & Bassingthwaighte, 2011). For example, MPC can take MML code representing a single tissue exchange region (26 lines), and generate a whole organ heterogeneous model for convection, diffusion, and reaction with 20 regions (1698 lines). See http://www.physiome.org/jsim/models/webmodel/NSR/MPC/. MPC currently runs outside of JSim but is planned for incorporation into a future JSim release.

**Modular construction with SemSim:** Precise semantic identification of variables and parameters is a prerequisite to merging of preconstructed submodels or modules into integrated systems or multiscale models. A future release of JSim will incorporate the tools for annotating models and their computational elements against biomedical ontologies and knowledge bases ( Rubin *et al.*, 2006). These annotations will make it easier for users to search the Physiome Model repository and to identify the biological phenomena modeled. Formatted according to the semantic simulation (SemSim) framework ( Gennari *et al.*, 2011), these annotations will also make it possible for tools to decompose and merge models in a more automated fashion, and allow the modeler to work at a biological, rather than computational level of abstraction ( Beard *et al.*, 2012). For example, selection of an ion pump, such as the NaKATPase, would bring up a set of modules from which the modeler would choose the version suited to the particular context, and then the code for the integrated model would be automatically generated from the annotated modules in the library.

### Getting started with JSim

Information for download and installation, running JSim, and writing JSim MML models can be found at http://www.physiome.org/jsim/. Software is also permanently available from: 10.5281/zenodo.7635.
