# Supplementary material for: JSim, an open-source modeling system for data analysis
Source: F1000Res. 2013 Dec 30;2:288. [Version 1] doi: 10.12688/f1000research.2-288.v1 (PMC3901508; doi:10.12688/f1000research.2-288.v1)
Supplement: Peer review history

# Peer review: Referee response for version 1

**Published:** 2014-01-23

**Authors:** David Nickerson

**Affiliations:** 1 Auckland Bioengineering Institute, University of Auckland, Auckland, New Zealand

This manuscript provides an introduction to, and description of, the JSim modelling system. The authors highlight the general purpose utility of this platform through the use of specific application examples, that are easily understood and followed by the reader and potential JSim users. This manuscript provides all appropriate links and examples that readers would require to get up and running with the JSim software.

This paper is well written, with just a few points that the authors may want to consider in future revisions of the manuscript.

- The authors touch briefly on reuse of existing models/projects and the use of a CVS repository to archive the history of model development, as well as the discussion on modular modelling in the future developments section. The basis for this modularity and reuse seems to result in the development of a new, monolithic MML document for the assembled model. It might be useful to see if there are features in either JSim or MML that allow dynamic links to the source modules to be maintained allowing users to alternate sources or versions of the source modules (rather than the cut-and-paste style described in the manuscript).
   
- In addition to the versioning of the JSim input data (experimental data, MML, projects, etc.), it is often the case that a specific piece of work requires some minimum version of the software itself. I wonder if there is any link between project files and JSim releases? For example, are users browsing the Physiome Repository able to determine if they need to update their version of JSim prior to loading a project file (or if in fact the software handles this internally).
   
- The manuscript would benefit from a more thorough comparison of JSim to alternative tools, or at least some links to specific tools being contrasted in the article.
   
- There is no description of how spatial geometries (finite element meshes or finite difference grids) are defined in JSim. Are the evolving standards for such descriptions (e.g., FieldML or SBML-spatial) being used or are there plans to use such? A comparison with approaches taken by tools like the Virtual Cell or Chaste might be useful.
   
- The authors make no reference to the adoption or interchange with the SED-ML standard. It would be useful to discuss any plans in this regard. Similarly, the evolving COMBINE archive format has a large overlap in aims with the JSim project file and the authors might want to comment on any plans to make use of that archiving format or contributions in that direction.
   
- In some parts of the manuscript (e.g., the caption for figure 3) the description of the modelling/simulation example seems a bit excessive, and detracts from the primary focus of the article.

**Minor comments**

- Page 5, column 1, first paragraph: Antimony is mentioned as a model import source format, but that format is not defined previously.
   
- Page 10, column 1, third paragraph: "...using several different numerical method," missing 's' on method.

I have read this submission. I believe that I have an appropriate level of expertise to confirm that it is of an acceptable scientific standard.

# Peer review: Referee response for version 1

**Published:** 2014-01-10

**Authors:** Steven Niederer

**Affiliations:** 1 Biomedical Engineering Department, King's College London, London, UK

The paper “JSim, an open-source modelling system for data analysis” provides a succinct update on the functionality and utility of the modelling platform JSim. The paper provides a concise description and link between the JSim community modelling philosophy and how this is facilitated by the JSim software platform. The complete description of the JSim environment will be of interest to the modelling community and this manuscript highlights much of the functionality that they require.  

This publication could be improved by addressing:

- As described in the article, the platform JSim has been developed over a period of over 40 years. Previous articles on JSim have been published and this article would be strengthened by highlighting the new features / functionality added to the platform since the previous JSim article.
   
- The article, as exemplified in the abstract, focuses on the technical functionality of JSim. If JSim can be readily set up or used by people who wish to analyse their experimental data with models, for example experimental researchers, as opposed to developing new models, then it would be worth highlighting this in the abstract and text.
   
- The article does not discuss or review alternate simulation platforms (for example COR, OpenCell, Continuity, CHASTE, SBML simulation environments). For new users wishing to make an informed decision it would be useful to highlight the differences between JSim and alternate platforms.

**Minor comments**

- In the introduction, the statement that mathematical models provide clear and precise hypothesis that are susceptible to contradiction and that failure to fit leads to rejection needs to be more nuanced, particularly in the case of biology where comparisons are often made between deterministic models and variable experimental results.
   
- It is not clear in the loop section if JSim supports nested loops, this could be clarified.
   
- It would be of interest to provide some indicative performance measures. For example if simulating a cardiac action potential will JSim solve faster or slower than real time on a conventional desktop.
   
- The authors could comment on the utility or potential for adoption of new mark-up languages for spatial problems (FieldML) or problem definition formats (SED-ML).
   
- It would be interesting for the authors to comment on how or if they have verified the JSim code stack.  

I have read this submission. I believe that I have an appropriate level of expertise to confirm that it is of an acceptable scientific standard.
